# Complementary and alternative medicine for functional dyspepsia: An Asian perspective

**DOI:** 10.1097/MD.0000000000030077

**Published:** 2022-09-02

**Authors:** Jin Young Yoon, Seok-Jae Ko, Jae-Woo Park, Jae Myung Cha

**Affiliations:** a Department of Internal Medicine, Kyung Hee University Hospital at Gangdong, Kyung Hee University School of Medicine, Seoul, Republic of Korea; b Department of Internal Medicine, Kyung Hee University Hospital at Gangdong, College of Korean Medicine, Kyung Hee University, Seoul, Republic of Korea.

**Keywords:** acupuncture, complementary and alternative medicine, functional dyspepsia, herbal medicine, psychotherapy

## Abstract

A considerable number of Asian patients with functional dyspepsia (FD) are searching for complementary and alternative medicine (CAM) because they are unsatisfied with conventional medical treatment. However, no article has focused on CAM for FD from Asian perspective. In Asian countries, many traditional herbal formulas, including *Banha-sasim-tang*, *Soyo-san*, *Sihosogan-san*, *Yukgunja-tang*, and *Pyeongwi-san*, are used in patients with FD. In the few blinded and placebo-controlled studies conducted, the weak evidence regarding the effectiveness of herbal prescriptions in patients with FD did not allow any conclusions to be made. The clinical efficacy and safety of STW-5 were proven in several prospective randomized controlled trials and systematic reviews. Hence, it was recently approved as a therapeutic option for the treatment of FD. Peppermint and caraway, FDgard, black seed oil, and Jollab have been used in patients with FD, but there is limited evidence supporting their use. Reviews of acupuncture in patients with FD showed inconsistent results: a Cochrane review reported a negative result, while other meta-analyses reported positive results. Psychotherapy, including hypnotherapy, psychoanalytic psychotherapy, and cognitive behavioral therapy, may be used in patients with FD, although it is only supported by weak evidence. Therefore, well-planned, large-scale studies are necessary to evaluate the efficacy of CAM in treating FD, especially in Asian countries.

## 1. Introduction

Functional dyspepsia (FD) comprises a heterogeneous group of symptoms arising from the epigastric region in the absence of any organic diseases that could explain these symptoms.^[1]^ Approximately 40% of patients with FD seek medical treatment.^[2]^ However, patients with FD remain unsatisfied with treatment as the most suitable medical treatment has not been established.^[1,3]^ Therefore, patients with FD often turn to complementary and alternative medicine (CAM) as a therapeutic alternative. These patients also desire a sense of control over their bodies and health.^[4]^ CAM comprises a unique group of medical practices and products that are not widely taught in medical schools and not generally available in public hospitals.^[5]^ It is particularly appealing to patients in whom conventional medical treatments fail or in cases where long-term pharmacological treatments are needed.^[4]^ CAM was used in 52.5% of gastroenterology outpatients in a Canadian study^[6]^ and 48.7% of patients with functional gastrointestinal (GI) disorders in an Italian survey.^[7]^ The prevalence of CAM use ranged from 23.6% to 44% in a systematic review (SR) of Australian and American adults with GI disorders.^[8]^ Despite the widespread use of CAM for functional GI disorders, no review article has covered CAM for FD from Asian perspective. Several herbal prescriptions and a combination of different traditional medicines, acupuncture, and psychotherapies have been proposed in different countries. In-depth knowledge and understanding of the use of CAM in patients with FD are needed to allow a better rapport with patients and to expatiate on the evidence-based consultations of CAM. This review intends to increase our knowledge and understanding of CAM, especially for patients with FD.

## 2. Main body

### 2.1. Traditional herbal medicine

Traditional herbal medicines have been used by various races in Asia, and they are still used by some physicians or as home remedies. Traditional medicines mainly target dysfunctional symptoms that are not confirmed by gross organic pathologies whereas Western medicine mainly targets organic pathologies identified by laboratory or radiologic examinations. From the perspective of traditional medicine, symptoms of FD are related to epigastric stuffiness, epigastric pain, or nausea.^[9]^ According to traditional Korean medicine (TKM), FD can be caused by disagreements between the liver and stomach, phlegm (retention of fluid) in the stomach, deficiency of stomach *qi*, *qi* deficiency or stagnation, blood stasis, food accumulation, or phlegm-fluid retention.^[10]^ However, it is difficult to define FD using these concepts as TKM uses a concept that cannot be explained in Western medicine. For example, the concept of the “stomach” used in TKM is not the same as the organ “stomach” in Western medicine,^[11]^ because the terminology of the “stomach” in TKM has a different physiological and pathological concept from internal organ “stomach” in Western medicine. Additionally, essential theories of TKM, such as *qi*, *yin-yang*, the 5 elements, or meridian (special points at the vessels of *qi*) have not been fully demonstrated in Western medicine.^[12]^ Fortunately, many recent studies evaluating the efficacy and safety of traditional herbal medicines have tried to use the Rome criteria, based on the concept of Western medicine, for the diagnosis of FD. The role of herbal prescriptions in the treatment of FD remains unclear. The mechanisms of action of these ingredients have not been fully identified, and well-designed randomized controlled trials (RCTs) are scarce. The standardization of herbal regimens and a clear identification of suitable applications through high-quality clinical evidence could help improve the role of CAM in treatment strategies.

#### 2.1.1. Traditional Korean medicine (TKM).

##### 2.1.1.1. *Banha-sasim-tang* (BST).

**Figure 1.**
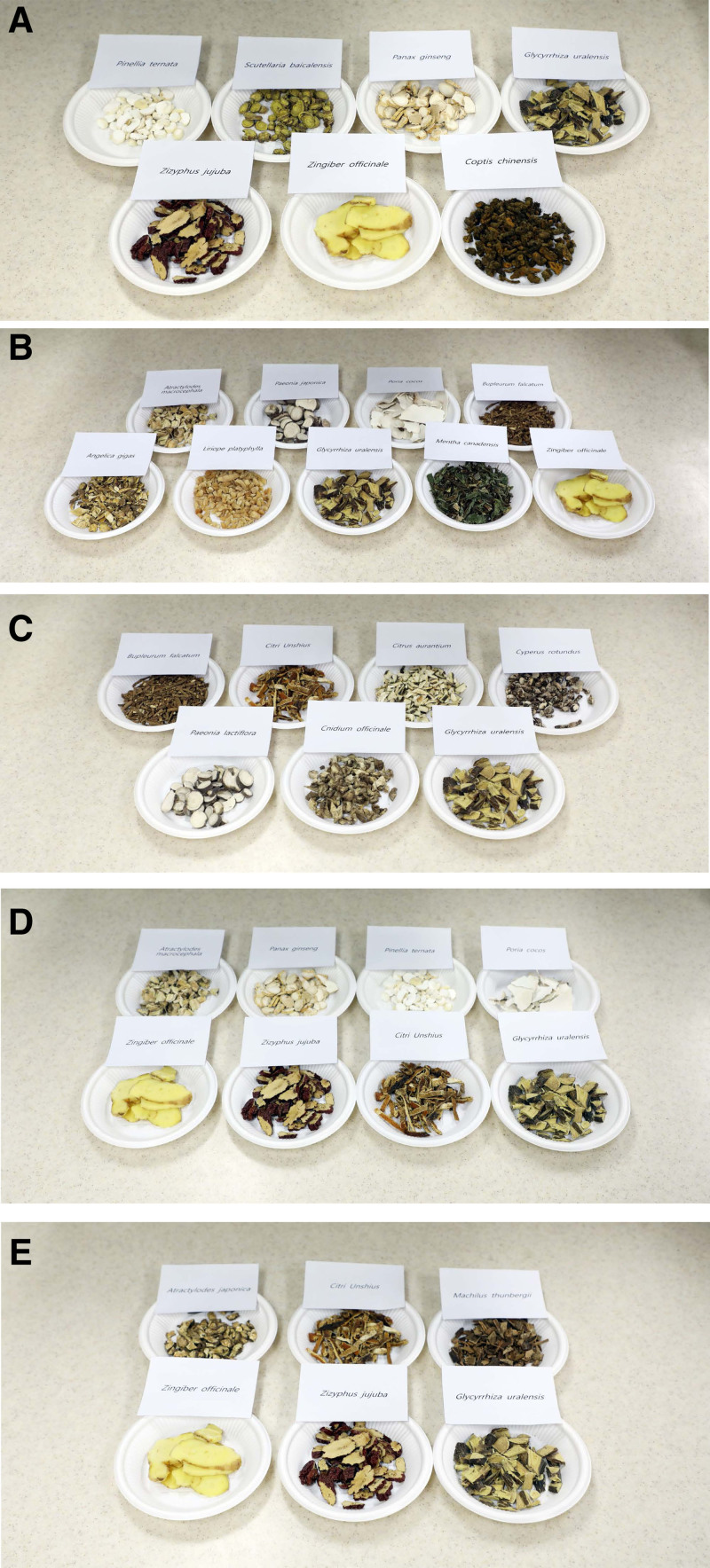
Herbal components of (A) *Banha-sasim-tang*, (B) *Soyo-san*, (C) *Sihosogan-san*, (D) *Yukgunja-tang*, and (E) *Pyeongwi-san* used in patients with functional dyspepsia.

BST, termed as *Banxia-xiexin-tang* in traditional Chinese medicine (TCM) and *Hange-shashin-to* in Kampo medicine (traditional Japanese herbal), is one of the herbal prescriptions used in TKM to treat epigastric stuffiness in patients with FD (Table 1).^[13]^ BST is composed of 7 herbs: *Pinellia ternata*, *Scutellaria baicalensis*, *Panax ginseng*, *Glycyrrhiza uralensis*, *Zizyphus jujuba*, *Zingiber officinale*, and *Coptis chinensis* (Fig. 1A).^[14]^ A Chinese RCT, including 67 patients in the treatment group and 34 in the placebo group, reported that the total dyspepsia symptom scale score improved significantly in the treatment group when compared with the placebo group after 4 weeks (65.9% vs 32.4%, *P* < .01).^[13]^ A Korean RCT recruited 100 FD patients receiving BST or a placebo for 6 weeks.^[14]^ Park et al^[14]^ found that there were no significant differences in the overall dyspeptic symptoms or quality of life (QoL) between the BST and placebo groups. Two SRs and 1 meta-analysis described the efficacy and safety of BST in the treatment of FD.^[15–17]^ A meta-analysis of 10 studies with a total of 972 patients compared the efficacy of BST and prokinetic agents and showed that BST had a better effect (odd ratio [OR] = 2.75, 95% confidence interval [CI] = 1.86–4.07) and a lower incidence of adverse events (AEs) than the prokinetics group in treating FD.^[15]^ However, this meta-analysis had a critical drawback: as RCTs were included in the meta-analysis regardless of blinding, none of the trials described allocation concealment or blinding. Moreover, except for 2 trials that clearly mentioned the randomization methods, the others did not describe the details of their randomization methods. Both SRs showed that BST was effective in treating FD. However, the treatment groups were administered heterogeneous treatment regimens consisting of other herbal prescriptions as well as BST.^[16,17]^ The poor methodological quality of the meta-analysis and SRs did not provide sufficient evidence to allow application of the guidelines for the treatment of FD patients. Recently, 1 group reported a well-designed study protocol for an SR of RCTs that focused on BST for the treatment of FD,^[49]^ but their results have not been reported yet. BST is a widely used TKM regimen for patients with FD; nevertheless, there is a paucity of credible evidence assessing its efficacy and safety in comparison with a placebo and other drugs used for FD in Western medicine.

**Table 1:** Complementary and alternative medicine used for patients with functional dyspepsia.

Traditional herbal medicine	Composition	Publications	Comment
Traditional Korean medicine			
* Banha-sasim-tang* (BST)	*Pinellia ternata*, *Scutellaria baicalensis*, *Panax ginseng*, *Glycyrrhiza uralensis*, *Zizyphus jujuba*, *Zingiber officinale*, *Coptis chinensis*	Chinese RCT,^[13]^ Korean RCT^[14]^ SRs/meta-analysis^[15–17]^	*Banxia-xiexin-tang* (TCM), *Hange-shashin-to* (Kampo medicine)
* Soyo-san* (SYS)	*Atractylodes macrocephala*, *Paeonia japonica*, *Poria cocos*, *Bupleurum falcatum*, *Angelica gigas*, *Liriope platyphylla*, *Glycyrrhiza uralensis*, *Mentha canadensis*, *Zingiber officinale*	Chinese RCT,^[18]^ animal study,^[19]^ Chinese meta-analysis^[20]^	*Xiaoyao-san* (TCM), *Shoyo-san* (Kampo medicine)
*Sihosogan-san* (SHS)	*Bupleurum falcatum*, *Citri Unshius*, *Citrus aurantium*, *Cyperus rotundus*, *Paeonia lactiflora*, *Cnidium officinale*, *Glycyrrhiza uralensis*	Chinese meta-analysis^[21]^	*Chaihu-shugan-san* (TCM), *Saikosokan-to* (Kampo medicine)
*Yukgunja-tang* (YGT)	*Atractylodes macrocephala*, *Panax ginseng*, *Pinellia ternata*, *Poria cocos*, *Zingiber officinale*, *Ziziphus jujuba*, *Citrus unshiu*, *Glycyrrhiza uralensis*	Korean meta-analysis,^[22]^ Japanese RCTs^[23,24]^	*Liu-jun-zi-tang* (TCM), *Rikkunshito* (Kampo medicine)
*Pyeongwi-san* (PWS)	*Atractylodes japonica*, *Citrus unshiu*, *Machilus thunbergii*, *Zingiber officinale*, *Zizyphus jujuba*, *Glycyrrhiza uralensis*	Animal study,^[25]^ Korean RCT^[26]^	
* Si-mo-tang* (SMT)	*Saussurea lappa*, *Citrus aurantium*, *Areca catechu Linn*, *Lindera aggregata* Kosterm	Chinese SR/meta-analysis^[27]^	
* Hangekoboku-to* (HKT)	*Pinellia ternata*, *Poria cocos*, *Magnolia denudata*, *Perilla frutescens*, *Zingiber officinale*	Japanese single-arm study^[28]^	
STW-5	*Angelica gigas*, *Silybum marianum*, *Carum carvi*, *Chelidonium majus*, *Iberis amara*, *Glycyrrhiza uralensis*, *Chamaemelum nobile*, *Mentha piperita*, *Melissa officinalis*	Multicenter, double-blind study,^[29]^ double-blind RCTs,^[30,31]^ Swiss meta-analysis,^[32]^ retrospective cohort study^[33]^	Iberogast (Bayer AG, Germany), developed in Germany in 1961
Other natural products	A combination of peppermint oil and caraway oil	Germany single-arm study,^[29]^ animal study,^[34]^ Chinese SR/meta-analysis^[35]^	
	l-Menthol and caraway	Germany RCT,^[36]^ American RCT^[37]^	FDgard, a commercially available medical food
	*N sativa* seed oil (black seed oil)	Irani double-blind RCT^[38]^	Traditional Persian medicine
	Jollab (combination of saffron, rose water, white rock candy, water, and others)	Irani double-blind RCT^[39]^	
Acupuncture		Korean RCT,^[40]^ meta-analysis,^[41–43]^ Cochrane review^[44]^	
Psychotherapy	Hypnotherapy	British RCT,^[45]^ Italian SR^[46]^	
	Psychoanalytic psychotherapy	British RCT,^[47]^ Irani controlled trial^[48]^	

RCT = randomized controlled trial, SR = systemic review, TCM = traditional Chinese medicine.

##### 2.1.1.2. *Soyo-san* (SYS).

SYS, termed as *Xiaoyao-san* in TCM and *Shoyo-san* in Kampo medicine, is used in patients with FD to treat abdominal pain and postprandial bloating.^[18]^ SYS is composed of 9 herbs: *Atractylodes macrocephala*, *Paeonia japonica*, *Poria cocos*, *Bupleurum falcatum*, *Angelica gigas*, *Liriope platyphylla*, *Glycyrrhiza uralensis*, *Mentha canadensis*, and *Zingiber officinale* (Fig. 1B).^[19]^ It has been shown to have an antidepressant-like effect in in vivo and clinical trials.^[18,19]^ Du et al^[18]^ reported the results of a double-blinded RCT that investigated the efficacy and safety of SYS in 180 perimenopausal women with FD and depression. After 8 weeks of treatment, the SYS group showed a significantly lower Hamilton Depression Rating Scale score (*P* < .01) and an improved gastric emptying rate (*P* < .05) when compared to the control group. SYS is expected to relieve FD symptoms associated with emotional disorders.^[18]^ However, these results should be interpreted with caution, as the primary endpoint of this study (improvement in the clinical symptoms and signs with ≥30% reduction in the accumulated score of symptoms) was not evaluated. Furthermore, this study focused only on women aged 41 to 52 years.

A Chinese meta-analysis of RCTs, including 14 studies with a total of 1338 patients, compared the efficacy of modified SYS and prokinetic agents in patients with FD.^[20]^ It showed that modified SYS had a better effect on symptom improvement than prokinetic agents (OR = 3.26, 95% CI = 2.24–4.47), without serious AEs. Furthermore, a better effect on symptom improvement was observed with the combination of modified SYS and prokinetic agents than with prokinetic agents alone (OR = 4.32, 95% CI = 2.64–7.08). This meta-analysis was limited as it was not blinded, and no placebo-controlled RCTs were included. Vague randomization was noted in most of the included studies, with no description of dropouts and withdrawals. Furthermore, diverse prokinetics with variable study durations (2–8 weeks) were used in the control group. The insufficient quality of the level of evidence does not allow conclusions to be made regarding the effectiveness of SYS. Therefore, there is a need to assess the effects of SYS in comparison with placebo and other drugs, with special attention to adequate randomization, blinding, sample size, placebo-control, and reporting of AEs. Recently, 1 group reported a well-designed study protocol for an SR of RCTs that focused on SYS for the treatment of FD, but its results have not yet been reported.^[19]^

##### 2.1.1.3. *Sihosogan-san* (SHS).

SHS, termed as *Chaihu-shugan-san* in TCM and *Saikosokan-to* in Kampo medicine, has been used in patients with FD.^[21]^ SHS is composed of 7 herbs: *Bupleurum falcatum*, *Citri Unshius*, *Citrus aurantium*, *Cyperus rotundus*, *Paeonia lactiflora*, *Cnidium officinale*, and *Glycyrrhiza uralensis* (Fig. 1C).^[21]^ A Chinese meta-analysis of RCTs, including 22 studies with a total of 1998 patients, compared the efficacy of modified SHS and prokinetic agents for the treatment of FD.^[21]^ The modified SHS had a better effect on symptom improvement when compared to the prokinetic agents (relative risk [RR] = 1.20, 95% CI = 1.14–1.27). Moreover, a better effect on symptom improvement was observed with a combination of modified SHS and prokinetic agents than with prokinetic agents alone (RR = 1.18, 95% CI = 1.11–1.25), with no serious AEs in either group. This meta-analysis showed that modified SHS alone or in combination with prokinetics might be more effective than prokinetics alone. However, all the studies were conducted in Chinese, and no randomization was performed, except in 2 trials, which weakened the reliability and repeatability of the research. The duration of the course of treatment varied from 3 weeks to 12 weeks, and the intention-to-treat analysis could not be performed because there were no follow-up records in most of the literature. These limitations could lead to a biased judgment regarding the efficacy of SHS, making it difficult to determine its actual role in the management of FD. Recently, 1 group reported a well-designed study protocol for an SR of RCTs that focused on SHS for the treatment of FD, but its results have not yet been reported.^[50]^

##### 2.1.1.4. *Yukgunja-tang* (YGT).

YGT, termed *Liu-jun-zi-tang* in TCM and *Rikkunshito* in Kampo medicine, has been prescribed for patients with FD to reduce dyspeptic or reflux symptoms and to improve fundic relaxation.^[22]^ YGT is composed of 8 herbs: *Atractylodes macrocephala*, *Panax ginseng*, *Pinellia ternata*, *Poria cocos*, *Zingiber officinale*, *Ziziphus jujuba*, *Citrus unshiu*, and *Glycyrrhiza uralensis* (Fig. 1D). A recent meta-analysis of 52 RCTs with a total of 5475 patients compared YGT with placebo, no treatment, or conventional Western medicine, including prokinetics, proton pump inhibitors, and antidepressants, for the treatment of FD.^[22]^ YGT showed significantly better results with a higher total clinical efficacy rate than Western medicine (RR = 1.21, 95% CI = 1.17–1.25; *P* < .001). YGT also showed a higher reduction in the total dyspepsia symptom score, greater improvement of gastric emptying rate, and lower recurrence for 6 months after treatment when compared to Western medicine. Furthermore, a combination therapy of YGT and Western medicine significantly improved the total clinical efficacy rate when compared with Western medicine alone (RR = 1.19, 95% CI = 1.14–1.24; *P* < .001). However, the authors of this meta-analysis interpreted that the overall methodological quality of the included studies was low owing to selection bias, performance bias, detection bias, and no reports on allocation concealment and blinding assessment. Additionally, they mentioned that only 5 of the 59 RCTs included in the qualitative synthesis were double-blinded, and 33 of 59 RCTs did not mention the occurrence of AEs. A multicenter, double-blind, placebo-controlled RCT in Japan evaluated the efficacy and safety of administering YGT (2.5 g) for 8 weeks to treat FD in 247 patients.^[23]^ At 8 weeks, the epigastric pain significantly improved (*P* = .04); however, the global patient assessment in the YGT group did not show a significant improvement when compared with the placebo group (*P* = .09). Interestingly, YGT was more effective than placebo in patients with *Helicobacter pylori* infection (40.0% vs 20.5%; *P* = .07) but less effective in those without infection (29.3% vs 25.6%; *P* = .72).^[23]^ The Japanese DREAM study, a multicenter, randomized, double-blind, placebo-controlled trial, showed that YGT significantly improved dyspeptic and psychological symptoms when compared to placebo in 128 FD patients without *H pylori* infection, diagnosed according to the Rome III criteria.^[24]^ The YGT group showed significant improvement when compared to the placebo group at 8 weeks. Furthermore, it not only reduces upper GI symptoms, especially postprandial fullness, early satiety, and bloating, but also anxiety. Notably, the improvements in psychological symptoms were related to those of the upper GI symptoms.^[24]^ Another study reported that YGT may be more useful in treating postprandial distress syndrome, a type of FD.^[51]^ YGT is one of the most popular and widely used formulas, which has been marketed to treat various GI symptoms, particularly in Japan.^[23,24,51,52]^ The Japanese Ministry of Health and Welfare has approved *Rikkunshito* for medical use (TJ-43, Tsumura & Co., Tokyo, Japan).^[52]^

##### 2.1.1.5. *Pyeongwi-san* (PWS).

PWS has been prescribed for the treatment of symptoms associated with the digestive system.^[53]^ It is composed of 6 herbs: *Atractylodes japonica*, *Citrus unshiu*, *Machilus thunbergii*, *Zingiber officinale*, *Zizyphus jujuba*, and *Glycyrrhiza uralensis* (Fig. 1E). A previous study demonstrated that PWS has a protective effect on the gastric mucosal lesion membrane. It not only induced anti-inflammatory effects and inhibitory mechanisms in macrophages, but also inhibited activity in vivo.^[25]^ An RCT including 170 patients with FD (86 patients in the PWS group and 84 in the placebo group) evaluated the Nepean Dyspepsia Index (NDI) and FD-QoL at 0, 4, and 8 weeks.^[26]^ The PWS group showed significant improvement in the FD-QoL measure, but not in the total symptom score of NDI when compared to the placebo group. However, this study was limited because the efficacy of PWS was not demonstrated for the clinical symptoms of FD (NDI).

##### 2.1.1.6. Other formulas.

*Si-mo-tang* (SMT),^[27]^ *Hangekoboku-to* (HKT),^[28]^ and *Zhizhu Kuanzhong* capsule^[54]^ have also been used in patients with FD. SMT is composed of *Saussurea lappa*, *Citrus aurantium*, *Areca catechu* Linn, and *Lindera aggregata* Kosterm.^[27]^ In an SR and a meta-analysis of 27 RCTs that included 2713 participants, SMT showed significant improvement in clinical efficacy on total analysis (RR = 1.14, 95% CI = 1.09–1.20) and subgroup analysis after the exclusion of different interventions (RR = 1.17, 95% CI = 1.13–1.21).^[27]^ HKT (*Banxia-houpo-tang*) is composed of *Pinellia ternata*, *Poria cocos*, *Magnolia denudata*, *Perilla frutescens*, and *Zingiber officinale*.^[28]^ The outcome of HKT treatment for 2 weeks was compared between 30 patients with FD and 20 healthy volunteers, and a significant improvement in the Gastrointestinal Symptom Rating Scale score was noted in the former. *Zhizhu Kuanzhong* capsule is composed of *Atractylodes macrocephala*, *Citrus aurantium*, *Bupleurum chinense*, and *Crataegus pinnatifida*.^[54]^ The efficacy and safety of *Zhizhu Kuanzhong* capsule in FD have not yet been reported.^[54]^ *Yijung-tang* (*Liujunzi* decoction) and *Jisilsobi-san* (*Zhishi-xiaopi-wan*) have also been used in TKM for patients with FD, but no published articles are available for these regimens.

#### 2.2.2. STW-5 (Iberogast).

STW-5 (Iberogast, Bayer AG, Germany) is a liquid preparation obtained from 9 herbal extracts: *Angelica gigas* (Garden angelica root), *Silybum marianum* (Milk thistle fruits), *Carum carvi* (Caraway fruits), *Chelidonium majus* (Greater celandine), *Iberis amara* (Bitter candy tuft), *Glycyrrhiza uralensis* (Liquorice root), *Chamaemelum nobile* (Chamomile flowers), *Mentha piperita* (peppermint herb), and *Melissa officinalis* (balm leaf).^[29]^ It was developed in Germany in 1961 and is available without prescription in Europe. It has been claimed to possess GI motility improvement, anti-inflammatory, antioxidative, and free radical-inhibiting properties, as well as reduced gastric acid secretion.^[55]^ The clinical efficacy of STW-5 has been proven in several prospective RCTs and meta-analyses. A double-blind, noninferiority RCT compared STW-5 with cisapride in 186 patients with FD for 4 weeks.^[30]^ In this trial, patients were randomly assigned to 1 of the 3 treatment arms and a placebo group: STW-5, n = 61; STW-5-II, n = 62; and cisapride, n = 63. STW-5 and STW-5-II showed equal effectiveness in improving dyspepsia-specific symptom scores when compared to cisapride treatment. A multicenter, double-blind, placebo-controlled RCT conducted in Germany evaluated the efficacy of STW-5 for 8 weeks in 315 patients with FD.^[31]^ In this trial, the improvement of the gastrointestinal symptom (GIS) score was significantly higher in the STW-5 group than in the placebo group (*P* < .05), regardless of *H pylori* infection.^[31]^ However, no primary efficacy parameters were used, except for the GIS score, and the percentage of responders (based on the improvement of the GIS score by ≥40%) was very high in the placebo group (78%). In a meta-analysis of 3 placebo-controlled RCTs evaluating the efficacy and safety of STW-5 in treating FD, Melzer et al^[32]^ found that STW-5 was more effective than placebo with respect to reducing the severity of the most problematic symptoms (OR = 0.22, 95% CI = 0.11–0.47), and the AEs were similar in both groups.^[32]^ In a multicenter, placebo-controlled, double-blind study with 103 patients with FD, diagnosed by Rome II criteria, the GIS score decreased after 4 weeks of STW-5 administration. Additionally, the percentage of treatment responders was higher with STW-5 than with the placebo (75% vs 54%; *P* = .03).^[29]^ However, no correlation was observed between symptom improvement and gastric half-emptying time as measured by the 13C-octanoic acid breath test.^[29]^A retrospective, epidemiological cohort study in 23 randomized centers, where both STW-5 and metoclopramide were used routinely, compared the efficacy and safety of both these drugs.^[33]^ There was no relevant difference in the median treatment duration, but more patients were symptom-free after STW-5 treatment than after metoclopramide treatment (71.6 vs 62.8%; *P* = .012), and AEs were documented only with the latter. Moreover, more physicians assessed STW-5 to be effective (*P* < .01) and very well tolerated (*P* < .001) when compared to metoclopramide. Based on these data, STW-5 was recently approved as a therapeutic option for the treatment of FD in November 2010 by the Korean Food and Drug Administration.

#### 2.2.3. Other natural products.

Peppermint is a perennial herb (*Mentha piperita*) that grows throughout Europe and North America, and most (>80%) peppermint herbs contain l-menthol as the active ingredient. Caraway (*Carum carvi*), a plant native to Western Asia, Europe, and North Africa, has been widely used in food products owing to its pleasant flavor and preservative properties. Peppermint oil obtained by steam distillation from the fresh leaves of peppermint and caraway oil, derived from caraway fruits, have been used as common natural products in the treatment of GI symptoms. It is known that a combination of peppermint oil and caraway oil may have a prokinetic effect and interact synergistically to attenuate post-inflammatory visceral hyperalgesia, which could have therapeutic benefits in FD patients.^[29,34]^ Recently, FDgard (IM Health Science), a commercially available medical food formulated for the dietary management of FD, has been developed. This commercial product with a fixed dose of l-menthol and caraway is available as solid-state, triple-coated, targeted-release microspheres. This combination showed a significant improvement in bloating and epigastric pain in patients with FD when compared to placebo.^[4,36]^ A SR and meta-analysis of the combination of peppermint and caraway in 5 RCTs with 578 participants showed that this combination had a statistically significant effect on the global improvement of FD symptoms (RR for “not much” or “very much” improvement = 0.59, 95% CI = 0.49–0.71) and improvement in epigastric pain (RR = 1.61, 95% CI = 1.28–2.03). The rate of AEs was similar in both groups.^[35]^ Unfortunately, this meta-analysis has several critical limitations of unreliable inclusion criteria due to the failure of diagnosis using Rome criteria, insufficient sample size for testing the publication bias, and a relatively short treatment duration of 4 weeks. Therefore, it should be interpreted with caution and confirmed by large-scale, long-term RCTs. A recent RCT compared the efficacy and safety of FDgard and placebo in 95 patients with FD at 24 hours and 4 weeks.^[37]^ It showed a statistically significant reduction in the FD symptoms in the treatment group than in the placebo group at 24 hours (*P* = .039), but a nonsignificant effect on the FD symptoms in patients with more severe symptoms after 4 weeks of treatment (*P* = .091). There was no statistically significant difference in the Global Overall Symptom scores and no serious AEs in either treatment group. The AEs of FDgard reported in other studies include diarrhea, nausea and vomiting, allergic contact dermatitis, urticaria, asthma exacerbation, and atrial fibrillation.^[4]^

In traditional Persian Medicine, black seed oil and Jollab have been used for FD.^[38,39]^ *Nigella sativa*, which is a small flowering plant that grows in Southwest Asia, Middle East, and Southern Europe, produces fruits with tiny black seeds. *N sativa* seed oil, commonly known as black seed oil, has been used in patients with FD.^[38]^ An Irani RCT including 70 patients with FD showed that the mean scores of the Hong Kong index of dyspepsia severity scores were significantly lower in the *N sativa* seed oil group than in the placebo group (*P* < .001) after 8 weeks of intervention, without any serious AEs. However, another RCT from Iran with 160 patients with FD showed that the Short Form of the Leeds Dyspepsia Questionnaire scores were significantly lower in the group using Jollab, a traditional beverage used for the treatment of FD, than in the placebo group after 4 weeks of intervention (*P* < .001).^[39]^

### 2.2. Acupuncture

**Figure 2.**
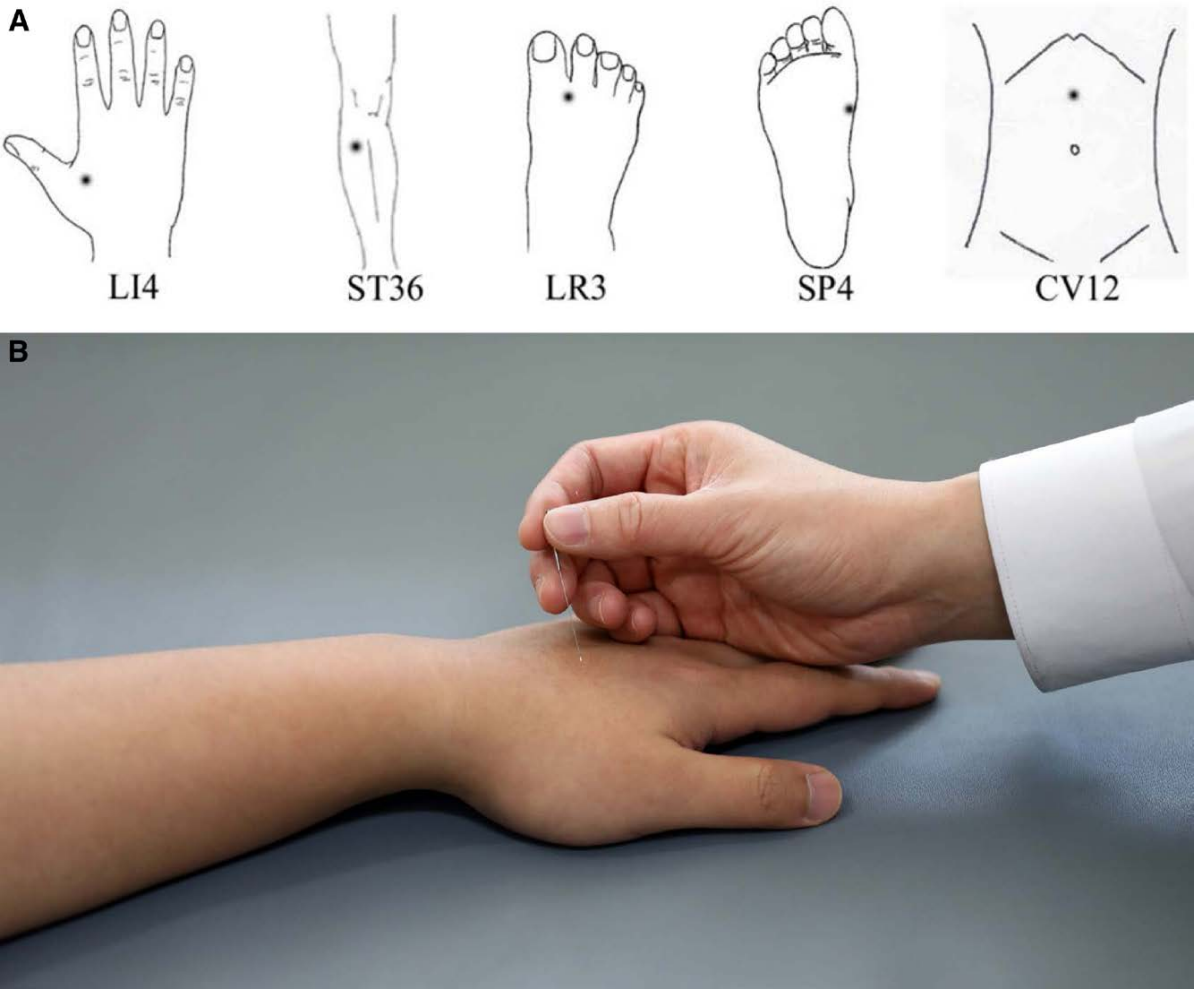
Acupuncture for treating functional dyspepsia; however, its scientific mechanism remains unclear. (A) Acupuncture point sites for treating patients with functional dyspepsia. (B) The acupuncture procedure (adapted from ref. ^[40]^, under authors’ agreement).

Acupuncture has been used as a traditional treatment in patients with GI disorders.^[40]^ It involves stimulating of certain acupuncture points by penetrating the skin with solid metallic needles, followed by manual manipulation of the needle, such as lifting, twisting, and thrusting (Fig. 2). Four recent meta-analysis studies evaluated the effectiveness of acupuncture in FD patients.^[41]^ The latest SR and network meta-analysis explored the most effective treatment between acupuncture and related therapies used alone or as an add-on to prokinetics and prokinetics used alone in 5 SRs with 22 RCTs.^[41]^ Two pairwise meta-analyses showed that manual acupuncture has a marginally stronger effect in alleviating global FD symptoms when compared to prokinetics (6 RCTs with domperidone: pooled RR = 1.21, 95% CI = 1.10–1.33, *P* = .0001) and 3 RCTs with itopride (pooled RR = 1.30, 95% CI = 1.11–1.52, *P* = .001). Additionally, it was found that a combination of manual acupuncture and clebopride had the highest probability of alleviating patient-reported global FD symptoms.^[41]^ A meta-analysis of 20 studies with 1423 patients showed that acupuncture was associated with a significant positive effect in patients with FD when compared to sham acupuncture (RR = 2.66, 95% CI = 1.85–3.82). Acupuncture also improved symptoms of FD better than prokinetics with respect to the total effective rate (RR = 1.18, 95% CI = 1.01–2.60).^[42]^ Another meta-analysis including 24 RCTs with 3097 patients showed that acupuncture significantly improved FD symptoms in studies reporting outcomes using dichotomous (RR = 1.19, 95% CI = 1.12–1.27) and continuous variables (standardized mean difference = −0.78, 95% CI = −1.21 to −0.35).^[43]^ Pooled analyses also showed that acupuncture improved the FD-related QoL (weighted mean difference = 5.97, 95% CI = 3.14–8.80) and health-related QoL (weighted mean difference = 6.83, 95% CI = 3.02–10.65), without serious AEs.

In contrast, a Cochrane review of 7 studies with 542 patients with FD concluded that acupuncture did not show better effects than prokinetics (cisapride, itopride, or domperidone) in 4 RCTs or sham acupuncture in 3 RCTs. These findings were independent of the acupuncture method, such as manual acupuncture, manual-electroacupuncture, or electroacupuncture.^[44]^ It also reported that acupuncture could lead to no or slightly more significant improvement in the scores measured by questionnaires such as the 36-Item Short-Form Health Survey, Self-Rating Anxiety Scale, the Self-Rating Depression Scale when compared with sham acupuncture. No statistically significant difference was reported between acupuncture and sham acupuncture in terms of AEs. However, no data for AEs were reported in studies examining manual acupuncture versus domperidone, manual-electroacupuncture versus domperidone, or electroacupuncture versus itopride. These inconsistent results may originate from the low quality of evidence level (no blinded trials, vague randomization, and short duration of follow-up) and inherent features of the acupuncture procedure (impossibility of complete blinding, high placebo effect, and operator dependence owing to no standardized protocol).

Serious AEs associated with acupuncture have not been reported in most RCTs. However, several AEs have been reported in relation to improper needle sterilization, resulting in infectious complications such as viral hepatitis, improper needle placement, and depth of insertion, which could cause solid and hollow organ perforation.^[4,56,57]^ Recently, however, sterilized disposable acupuncture needles are used; therefore, the occurrence of infectious complications is rare.^[58]^ The risk of AE incidence may be lower with TKM physicians because they not only learn basic medical sciences such as human anatomy, physiology, and pathology, but also have knowledge about diseases and Western medicine practice when compared to acupuncturists, who have limited education and knowledge in these fields.^[59]^ Therefore, the safety issue of acupuncture should be considered in real clinical practice considering diverse practitioners, patient populations, and variable proficiency in acupuncture.

### 2.3. Psychotherapy

The basis of CAM is a holistic approach wherein all diseases result from disturbances in the physical, psychological, social, and spiritual levels.^[4]^ Therefore, psychological therapies including hypnotherapy, psychoanalytic psychotherapy, and cognitive behavioral therapy have been used to ameliorate FD symptoms. Hypnotherapy has been used to manage patients with GI disorders because functional brain imaging demonstrated the involvement of sensorimotor and cognitive processes in hypnosis, a state of consciousness with concentrated attention, increased responsiveness to suggestion, and decreased peripheral awareness.^[60]^ Hypnotherapy was more effective (59%) than medical treatment (41%) (*P* = .01) or supportive therapy (33%) (*P* = .057) in FD patients in the short term (16 weeks). Additionally, it also significantly improved symptoms (73%) when compared with medical (20%) (*P* < .01) or supportive treatment (34%) (*P* < .01) in the long term (56 weeks).^[45]^ Furthermore, the QoL showed more significant improvement with hypnotherapy than with medical treatment.^[45]^ An SR including 4 of 398 articles reported a general improvement in the physical and mental health with hypnotherapy in patients with FD.^[46]^

Psychoanalytic psychotherapy is a method for changing or assessing relationship patterns through the improvement of interpersonal conflicts, emotional lability, and alexithymia.^[61]^ Hamilton et al^[47]^ showed that 73 FD patients, in whom conventional pharmacologic treatments had failed, presented significant improvements in the total symptom score with psychoanalytic psychotherapy when compared with a control group undergoing supportive therapy at 1 year (*P* = .015). Dehghanizade et al conducted 10 sessions of cognitive behavioral stress management or no intervention in 2 groups of 15 FD patients each. They found that cognitive behavioral stress management strategies were more effective in reducing symptoms when compared to the no intervention method.^[48]^

Psychotherapy is safe and effective in refractory cases and in those with extraintestinal symptoms and reduces the need for medication. Therefore, it could be an alternative effective technique for the treatment of patients with FD. However, psychotherapy has several limitations, as it is time-intensive, relatively expensive, and requires trained therapists and highly motivated patients. Furthermore, real-world data supporting the effectiveness of FD treatment are not sufficient to be applied to new guiding principles for treatment from a therapeutic perspective.

### 2.4. Limitations of complementary alternative medicine

Despite positive results in CAM trials for treating FD patients, the evidence for the CAM approach remains weak for several reasons. First, traditional herbal prescriptions are prescribed as mixtures of dried herbs that are decocted with water and have unique flavors in each combination. This makes it difficult to manufacture a placebo that is similar to the study medication, and thus, conducting a completely blinded test is almost impossible.^[62]^ Therefore, researchers hesitate to plan blinded RCTs, and most previous studies were limited by the methodological quality. In a few recent RCTs, however, the methodological quality has improved as granular herbal prescriptions were used instead of liquid herbal prescriptions with identical placebos.^[14]^ Second, most SRs included diverse prokinetic agents with variable treatment durations in the control group. Additionally, most RCTs used only prokinetics as a control treatment while evaluating the efficacy of FD, although proton pump inhibitor is one of the first-line treatments for FD. Third, research to date has been far too heterogeneous in terms of the study population due to unclear diagnostic criteria, intervention type due to various dosages and mixtures of herb prescriptions, and treatment duration. Furthermore, herbal medications used in Asian regions cannot be generalized across Western countries because very few well-designed RCTs have enrolled the Western population. Recently, several clinical trials focusing on patients with FD showed greater rates of placebo responses than those focusing on patients with organic GI diseases.^[63]^ A Cochrane review published in 2006 showed an average placebo response of 56% among FD patients.^[9]^ The rate of placebo response to conventional treatment in a meta-analysis with 45 FD trials varied from 31% to 45% and that of herbal medicine in a meta-analysis with 19 FD trials was 49%.^[17,64]^ In CAM for FD patients, the main question regarding placebo has been whether symptom improvement with a particular therapy was higher than that with placebo. Furthermore, the lack of a placebo arm in clinical trials involving acupuncture and psychotherapy weakened their evidence levels. The weak evidence due to the clinical trial design, not considering bias due to high placebo effects in patients with FD, does not allow us to assess the therapeutic effect objectively. Therefore, no conclusion regarding their effectiveness has been established, and only a few of the studies were ideally suited to investigate the effectiveness of herbal prescriptions in patients with FD.^[14]^ Recently, Chiarioni et al^[65]^ reviewed for the CAM in FD, such as peppermint oil or STW-5, from Western perspective, but little focused on TKM, TCM, or Kampo medicine, which were covered in detail from Asian perspective in the current review.

## 3. Conclusion

Physicians have perceived that CAMs lack scientific evidences, but, their evidences are accumulating in recent years from clinical researches on CAM. Physicians often dismiss CAM for patients with FD, but the potential synergistic combination of CAM and Western medicine should be explored in future studies with optimal methodological quality.

## Author contributions

Study concept and design: Jae Myung Cha

Drafting of the manuscript: Jae Myung Cha, Jin Young Yoon

Acquisition of data and provision of technical support: Jin Young Yoon, Jae-Woo Park, and Seok-Jae Ko

**Conceptualization:** Jae Myung Cha, Conceptualization: Jae-Woo Park.

**Data curation:** Seok-Jae Ko.

**Formal analysis:** Jin Young Yoon.

**Investigation:** Jin Young Yoon.

**Resources:** Seok-Jae Ko.

**Supervision:** Jae Myung Cha.

**Writing – original draft:** Jae Myung Cha, Jin Young Yoon.

**Writing – review & editing:** Jae Myung Cha, Jae-Woo Park.
